# Effect of decompressive hemicraniectomy on mortality of malignant middle cerebral artery infarction

**Published:** 2010

**Authors:** Bahram Aminmansour, Majeed Rezvany, Davood Sharifi, Hamidreza Shemshaki

**Affiliations:** a Associate Professor of Neurosurgery, Department of Neurosurgery, Isfahan University of Medical Sciences, Isfahan, Iran; b Assistant Professor of Neurosurgery, Department of Neurosurgery, Isfahan University of Medical Sciences, Isfahan, Iran; c Resident of Neurosurgery, Department of Neurosurgery, Isfahan University of Medical Sciences, Isfahan, Iran; d Research Assistant, Medical Students Research Center, Isfahan University of Medical Sciences, Isfahan, Iran

**Keywords:** Hemicraniectomy, MCA Infarction, Malignant Infarction, Mortality

## Abstract

**BACKGROUND:**

Increasing intracranial pressure (ICP) is one of the leading causes of mortality in patients with malignant infarction of the middle cerebral artery (MCA). We prospectively evaluated patients with MCA infarction for one month survival after decompressive hemicraniectomy.

**METHODS:**

This study was conducted at Alzahra University Hospital, Isfahan (Iran). Twenty patients with infarction in total MCA distribution area, resulting in midline shift of brain tissue for greater than 5mm, underwent decompressive hemicraniectomy. Mortality rate was estimated one month after surgery.

**RESULTS:**

Patients were 8 (40%) males and 12 (60%) females with a mean age of 49.9 ± 3.8 (25 to 70) years. Left and right MCA were involved in 7 (35%) and 13 (65%) patients, respectively. Four (20%) patients died within one month after surgery (3 females and one male, mean age of 59.0 ± 4.5 vs. 47.6 ± 3.4 in survived patients, p < 0.001). The mean of baseline Glasscow Coma Scale (GCS) score estimated 8.60 ± 1.55 in survived patients and 6.75 ± 0.95 in patients who died (p < 0.05).

**CONCLUSIONS:**

The survival rate of malignant MCA infarction treated with decompressive hemicraniectomy was the same as previous reports. MCA infarction mortality increased with age and lower admission GCS score.

Stroke in the middle cerebral artery (MCA) or its branches, leading to acute focal neurological deficits, is the most common type of anterior circulation infarcts. It is responsible for about 90% of infarcts and about 70% of all first strokes. The incidence of MCA infarction has been reported approximately 80 cases per 100,000 people per year.^1^ A recent systematic review by Feigin and colleagues on incidence of stroke reported that change in stroke incidence in different populations from 2000 to 2008 has partially been related to socio- economic status. It also showed a 42% decrease of stroke incidence in high- income countries while there was more than 100% increase in the countries with low to middle income. Overall, stroke incidence was 20% higher in low- to middle- income countries compared to high- income ones.^2^

Death due to acute ischemic stroke happens within the first 30 days after the attack in up to 30% of the cases and the hemorrhagic stroke has poorer prognosis with only a 20% survival rate. Death usually occurs from progressive swelling of the ischemic brain tissue, brain tissue shifting, increase in intracranial pressure (ICP), and the extension of ischemia to adjacent vascular territories.^3^,^4^ In patients with space- occupying hemispheric infarction, decreasing of ICP with surgical decompression within 48 hours from stroke onset has been shown to reduce patients’ mortality and improve functional outcome.^5^ The present report describes survival of patients with malignant MCA infarction who received decompressive hemicraniectomy.

## Methods

This prospective study was conducted from January 2008 to Jan 2010 in the Department of Neurosurgery at Al- Zahra University Hospital, Isfahan (Iran). The Ethics Committee of the Isfahan University of Medical Sciences approved the study protocol and informed consent was obtained from all patients’ family after full explanation of the study aims and protocol. After acquiring demographic data, such as age and gender and neurological examination, patients who had infarction in total MCA distribution area, resulting in brain tissue midline shifting for more than 5 mm on the brain Computed Tomography (CT) scan, underwent decompressive hemicraniectomy with the following technique.

Surgical technique- The bone was removed from one side of the skull measuring roughly 13 cm in the antero- posterior dimension, and from the floor of the middle cranial fossa to at least 9 cm superiorly, while simultaneously opening of dura. Cruciate or circumferential durotomy performed over the entire region of bony decompression to insure that nothing resists the expanding brain from being able to herniate outward. No brain resection or ventriculostomy was required. The bone removed during hemicraniectomy was saved in the bone bank in standard protocol and was then replaced after the swelling has subsided after 6 to 12 weeks.

Mortality rate estimated one month after hemicraniectomy. Independent sample t test and Chi square test were used for statistical analyses and a p value of less than 0.05 was considered statistically significant. Analyses were done using SPSS for windows (version 16.0).

## Results

The participants consisted of 8 (40%) males and 12 (60%) females with a mean age of 49.9 ± 3.8 years (25 to 70 years). Left MCA was involved in 7 (35%) patients and right MCA in 13 (65%) patients. Four (20%) patients died within one month **after** after surgery (3 females and one male). As presented in table 1, there was no significant difference between genders in survival rate (p > 0.05). The mean of admission Glasscow Coma Scale (GCS) score estimated 8.60 ± 1.55 in survived patients and 6.75 ± 0.95 in dead patients (p < 0.05). The mean age of dead patients after hemicraniectomy was 59.0 ± 4.5 compared with 47.6 ± 3.4 years in survived patients (p < 0.001).

**Table 1:** Comparison between patients who survived and those who died

	Survived within one month		P
	Yes, n = 16	No, n = 4	
Age	47.6 ± 3.4	59.0 ± 4.5	< 0.001*
Male/Female	7 (87.5%)/9 (75%)	1 (12.5%)/3 (25%)	0.291**
Baseline GCS score	8.60 ± 1.55	6.75 ± 0.95	0.036*

Data are presented as mean ± SD or number (%)

* Independent sample t test

** Chi square test

## Discussion

MCA infarction is a devastating form of ischemic attack with the mortality rate of 15- 30% at first month.^4^ Because of high rate of mortality and morbidities, detecting the causes of death and preventing or treating them is very necessary. Decompressive hemicraniectomy is a surgical method for decreasing ICP which has been used at first for sub arachnoid hemorrhage.^6^ Jüttler et al showed reduced hospital mortality from 60- 100% to 0- 29% and long- term mortality from 83- 100% to 33%^7^ in this regard. In another study, Gupta et al reported 24% mortality rate in patients with MCA infarction after 7- 12 months.^8^ The present results showed higher mortality was related to increasing age and lower GCS scores. These results were agreed to those of Rabinstein et al study which showed low admission GCS score, midline shift, presence of anisocoria, early clinical deterioration, and internal carotid artery occlusion as the preoperative predictors for patient’s outcome.^9^ However, in another study only age was the predictor for patients’ outcome.^8^ Several studies showed that patients with higher age had poorer survival.^10^,^11^ In the present study, the main etiology of death was related to metabolic disturbances or other organ failures within one month after hemicraniectomy and no patient died because of structural lesion.

There are some limitations to this study. To determine the morbidity after hemicraniectomy, it was useful to measure functional status of the patients after surgery and also in follow- ups. Also, for a thorough analysis of risk factors of mortality such as baseline characteristics, concurrent diseases of the patients, and interval between admission to surgery, a larger sample size is needed.

## Conclusions

In summary, the survival rate of malignant MCA infarction treated with decompressive hemicraniectomy in our center was the same as previous reports. Results also indicated that mortality rate associated with MCA infarction increased with age and lower GCS score at the time of admission. Further studies with larger sample sizes are recommended for evaluation of other prognostic factors.
